# 3-De­oxy-1,2-di-*O*-isopropyl­idene-5-*O*-tosyl-d-*threo*-pentofuran­ose

**DOI:** 10.1107/S1600536812010884

**Published:** 2012-03-17

**Authors:** Bogdan Doboszewski, Maria J. e Silva, Alexander Y. Nazarenko, Victor N. Nemykin

**Affiliations:** a Departamento de Química, Universidade Federal Rural de Pernambuco, 52171-900 Recife, PE, Brazil; b Departamento de Farmácia, Universidade Federal do Rio Grande do Norte, 59010-180 Natal, RN, Brazil; c Chemistry Department, State University of New York, College at Buffalo, 1300 Elmwood Ave, Buffalo, NY 14222-1095, USA; d Department of Chemistry & Biochemistry, University of Minnesota Duluth, Duluth, Minnesota 55812-2496 USA

## Abstract

In the crystal structure of the title compound, C_15_H_20_O_6_S, the two independent mol­ecules crystalllize in a chiral setting with two different conformations, twisted ^4^ *T*_3_ and envelope ^4^ *E*, for the furan­ose rings. Weak C—H⋯O contacts strengthen the crystal structure.

## Related literature

For the syntheses of this and similar compounds, see: Cox *et al.* (1997 ▶); Dahlman *et al.* (1986 ▶); Doboszewski & Herdewijn (1996 ▶, 2008 ▶). For conformations of five-membered rings, see: Cremer & Pople (1975 ▶); Boeyens & Dobson (1987 ▶). For weak C—H⋯O contacts, see: Desiraju & Steiner (1999 ▶). For analysis of absolute structure, see: Flack (1983 ▶); Hooft *et al.* (2008 ▶); Tipson (1944 ▶); Fieser & Fieser (1967 ▶) describe tosyl­ation reactions. For standard bond length data, see: Allen (2002 ▶).
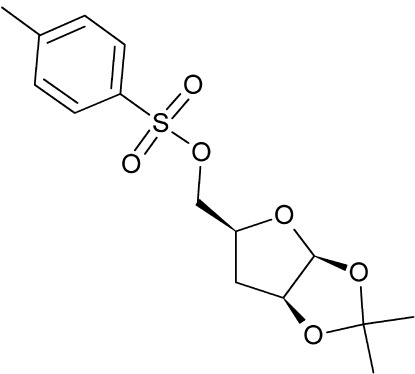


## Experimental

### 

#### Crystal data

C_15_H_20_O_6_S
*M* *_r_* = 328.37
Monoclinic, 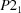

*a* = 10.9397 (1) Å
*b* = 9.4251 (1) Å
*c* = 15.4833 (10) Å
β = 96.414 (7)°
*V* = 1586.46 (10) Å^3^
*Z* = 4
Cu *K*α radiation
μ = 2.06 mm^−1^
*T* = 123 K
0.2 × 0.2 × 0.18 mm

#### Data collection

Rigaku R-AXIS RAPID II imaging plate diffractometer
Absorption correction: multi-scan (*ABSCOR*; Higashi, 1995 ▶) *T*_min_ = 0.55, *T*_max_ = 0.65
14179 measured reflections
4917 independent reflections
4440 reflections with *I* > 2σ(*I*)
*R*_int_ = 0.037

#### Refinement

*R*[*F* ^2^ > 2σ(*F* ^2^)] = 0.034
*wR*(*F* ^2^) = 0.087
*S* = 1.04
4917 reflections
404 parameters
1 restraint
H-atom parameters constrained
Δρ_max_ = 0.30 e Å^−3^
Δρ_min_ = −0.26 e Å^−3^
Absolute structure: Flack (1983 ▶), 2059 Friedel pairs
Flack parameter: 0.005 (12)

### 

Data collection: *CrystalClear-SM Expert* (Rigaku, 2009 ▶); cell refinement: *HKL-2000* (Otwinowski & Minor, 1997 ▶); data reduction: *CrystalClear-SM Expert*; program(s) used to solve structure: *SHELXS97* (Sheldrick, 2008 ▶); program(s) used to refine structure: *SHELXL97* (Sheldrick, 2008 ▶); molecular graphics: *ORTEP-3 for Windows* (Farrugia, 1999 ▶) and *Mercury* (Macrae *et al.*, 2008 ▶); software used to prepare material for publication: *PLATON* (Spek, 2009 ▶).

## Supplementary Material

Crystal structure: contains datablock(s) global, I. DOI: 10.1107/S1600536812010884/zl2463sup1.cif

Structure factors: contains datablock(s) I. DOI: 10.1107/S1600536812010884/zl2463Isup2.hkl

Supplementary material file. DOI: 10.1107/S1600536812010884/zl2463Isup3.cdx

Additional supplementary materials:  crystallographic information; 3D view; checkCIF report

**Fig. 5.**
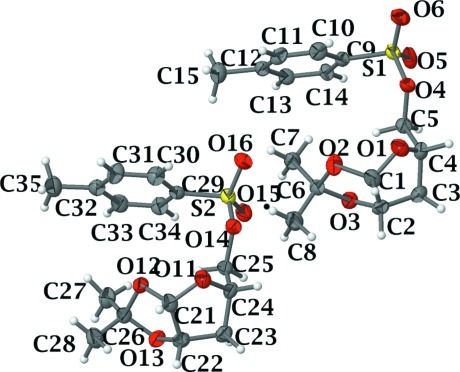
View of the title compound showing displacement ellipsoids at the 50% probability level.

Enhanced figure: interactive version of Fig. 5

## Figures and Tables

**Table 1:** Hydrogen-bond geometry (Å, °)

*D*—H⋯*A*	*D*—H	H⋯*A*	*D*⋯*A*	*D*—H⋯*A*
C4—H4⋯O5^i^	1.00	2.48	3.390 (3)	152
C5—H5*B*⋯O3	0.99	2.56	3.196 (3)	122
C11—H11⋯O12^ii^	0.95	2.44	3.163 (3)	133
C24—H24⋯O15^iii^	1.00	2.42	3.315 (3)	148
C28—H28*C*⋯O6^iv^	0.98	2.54	3.471 (3)	159

Symmetry codes: (i) 
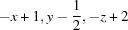
; (ii) 
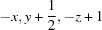
; (iii) 
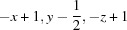
; (iv) 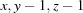.

**Fig. 1.**
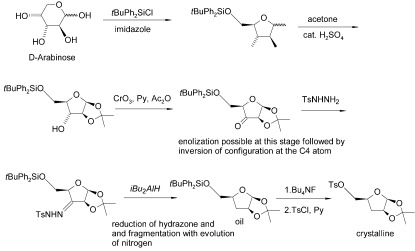
Scheme of the synthetic route leading to title compound.

**Fig. 2.**
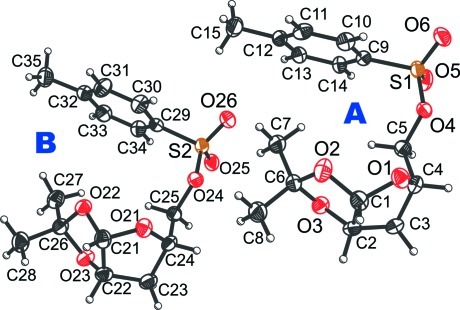
ORTEP view of two independent 3-deoxy-1,2-di-O-isopropylidene-5-O-tosyl-D-threo-pentofuranose molecules (A and B) with displacement ellipsoids drawn at the 50% probability level.

**Fig. 3.**
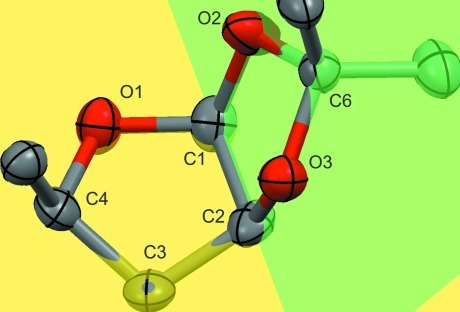
Twisted conformations of the five-membered rings in molecule A. Planes are drawn through atoms O1, C1, and C2 (yellow) and O2, C1, and C2 (green).

**Fig. 4.**
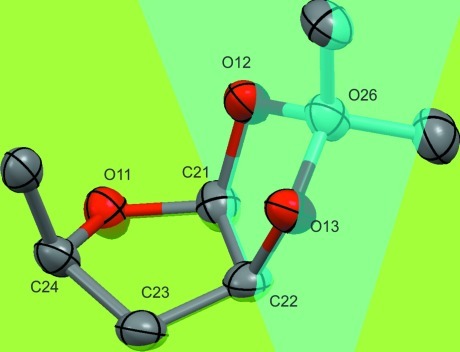
Envelope conformations of the five-membered rings in molecule B. Mean planes through atoms O11, C1, C2, C4 (yellow) and C1, C2, O2, and O3 (green).

## supplementary crystallographic information

### Comment

*D*- and *L*-arabinose are very convenient chiral-pool substrates for stereoselective synthesis since both of them are commercially available, reasonably priced, and easy to functionalize in two steps to form 5-*O*-*t*-butyldiphenylsilyl-1,2-*O*-isopropylidene furanose or its *L*-enantiomer (Dahlman *et al.*, 1986; Doboszewski & Herdewijn, 2008). Both enantiomers have been previously used in the synthesis of degradation products of the antibiotic Batumin/Kalimantacin A (Doboszewski & Herdewijn, 2008), to obtain branched-chain pyranosyl nucleosides (Doboszewski & Herdewijn, 1996) and *C*-hydroxymethylpentose present in lipopolysaccharides of *Coxiella brunetii (*Dahlman *et al.*, 1986), among others. Our current interest in arabinose stems from a possibility to convert it into the general substrates 3-deoxy-1,2-di-*O*-isopropylidene-5-*O*-tosyl-*D*-*threo*-pentofuranose and 3-deoxy-1,2-di-*O*-isopropylidene-5-*O*-butyldiphenylsilyl-*D*-*threo*-pentofuranose to be used in further transformations. A synthesis scheme for both these compounds is shown in Figure 1. We wanted to firmly establish their structures, due to a possibility of enolization of the ulose and concomitant inversion of configuration at the C4 position during formation of the tosylhydrazone.

A correct absolute structure of the title compound was important for the further synthetic work. Because of that, we have selected Cu *K*α radiation to ensure unambigous determination of the absolute structure.

In the crystal structure of the title compound (Fig.2), there are two crystallographically independent molecules, A (C1–C15, O1–O6, S1) and B (C21–C35, O11–O16, S2), in which all bond lengths and bond angles have standard dimensions. The six-membered phenyl rings in both molecules are flat within 0.01 Å.

It is visually obvious (Fig. 3 and Fig. 4) that the conformations of the five-membered rings differs in the two independent molecules A and B. A quantitative analysis of the ring conformations was performed using the method of Cremer and Pople (Cremer & Pople, 1975; Boeyens & Dobson, 1987) for the calculation of parameters of puckering. In molecule A, the polar parameters for the furanose ring and adjacent five membered ring are Q = 0.289 (3) and 0.312 (2) Å, Φ = 122.9 (5)° and 119.7 (5)°, respectively. These suggest a twisted ^4^T_3_ conformation for the furanose ring (ideal Φ = 126°), slightly distorted towards envelope (Φ = 108°). The substituent ring also has a twisted conformation (Fig. 3).

In molecule B (Fig. 4), the polar parameters for the furanose ring and the corresponding five membered ring are Q = 0.292 (3) and 0.361 (2) Å, Φ = 142.1 (5)° and 143.9 (4)°. These suggest an envelope conformation (ideal Φ = 144°) for both rings, with atoms C(24) and C(26) in the corners of the respective envelopes (^4^E for the furanose ring).

In the structure of 1,2-di-*O*-isopropylidene-5-*O*-tosyl-*D*-xylofuranose which differs from the title compound in one hydroxy group, the polar parameters are Q = 0.352 (3) Å, Φ = 288.8 (5)°; see refcodes RUWDES and RUWDES01 (Cox *et al.*, 1997). This makes the conformation an almost exact ^3^E envelope, but with a different carbon atom in the corner than in the case described here. Obviously, the furanose ring conformation is highly flexible and is easily influenced even by weak intermolecular interactions.

A short intramolecular contact is present between sulfonyl O atoms O5 and O15 and neighboring hydrogen atoms of the adjacent respective phenyl rings (see Table 1). This is quite common for aryl sulfonyls and the majority of these compounds exhibit these intramolecular interactions (mean H···O distance is 2.533 Å for more than 2500 analogous structures listed in the Cambridge Structural Database (Allen, 2002)). It may additionaly stabilize the conformation of the molecule. Only weak intermolecular C—H···O contacts (Table 1) exist between neighboring molecules.

### Experimental

Title compound was obtained as a product of a multi-step synthetic procedure (Doboszewski & Herdewijn, 2008; see Fig. 1). The tosylation of the previously synthesized 3-deoxy-1,2-di-*O*-isopropylidene-5-*O*-t-butyldiphenylsilyl-*D*-*threo*-pentofuranose by tosyl chloride in dry pyridine following standard reaction conditions (Tipson, 1944; Fieser & Fieser, 1967) produced the title compound in quantitative (near 100%) yield. Crystals suitable for X-ray diffraction experiment were crystallized from a hexane - diethyl ether mixture.

*R*_f_ 0.36 in hexane- EtOAc 2:1; mp. 346–348 K (from Et_2_O-hexane); α_D_ +42.8° (c 1.6 g/100mL, CHCl_3_); exact mass (electrospray): calc. for C_15_H_20_O_6_S + Na^+^= 351.0873, found 351.0872; ^1^H NMR (300 MHz, CDCl_3_): 7.80(d, J=8.3 Hz,2*H*), 7.34(d, J=8.3 Hz, 2H), 5.76(d, J=3.7 Hz, 1H), 4.69(t, J=4.6 Hz, 1H), 4.36(dddd, J=2.0 Hz, 6.5 Hz, 6.5 Hz, 8.4 Hz, 1H), 4.19(dd, J= 6.9 Hz, 9.7 Hz, 1H), 4.11(dd,J=6.6 Hz, 9.7 Hz, 1H), 2,44(s, 3H), 2.17(ddd, J=5.7 Hz, 8.5 Hz, 14.4 Hz, 1H), 2.04(dd, J=1.6 Hz, 14.5 Hz, 1H), 1.33 and 1.25(two s, 3H each); ^13^C NMR (75 MHz, CDCl_3_): 145.01, 132.96, 129.99, 128.16, 112.32, 106.94, 80.33, 77.91, 71.34, 33.68, 26.74, 25.70, 21.73. FTIR (diamond ATR): 2985, 2944, 1598, 1381, 1188, 991, 953, 705, 574 cm^-1^.

### Refinement

Final refinement was performed using TWIN/BASF type resulting in BASF = 0.00458. Analysis of the absolute structure using likelihood methods (Hooft *et al.*, 2008) was performed using *PLATON* (Spek, 2009); 2059 Bijvoet pairs were employed. The results confirmed that the absolute structure had been correctly assigned: the probability that the structure is inverted and probability of racemic twinning being statistically zero. All H atoms were positioned geometrically with C—H =0.95–1.00 Å and *U*_iso_(H) = 1.2 or 1.5 *U*_eq_(C). Rotating group refinement (AFIX 137) was employed for all methyl groups.

At data processing, a number of unobserved high angle reflections (with k from 8 to 11) of statistically zero intensity were excluded: 1 8 3, 2 8 3, 2 9 1, 3 9 0, 3 9 1, 3 9 2, 0 10 0, 0 10 1, 0 10 2, -5 10 2, -4 10 1, -4 10 2, -3 10 1, -2 10 1, -1 10 2, 1 10 0, 1 10 1, 1 10 2, 2 10 0, 3 10 0, 3 10 3, 4 10 0, 4 10 1, 4 10 2, 4 10 3, 5 10 2, -2 11 1, -3 11 1, 0 11 3.

### Crystal data

**Table:**

C_15_H_20_O_6_S	*F*(000) = 696
*M**_r_* = 328.37	*D*_x_ = 1.375 Mg m^−^^3^
Monoclinic, *P*2_1_	Melting point: 347(1) K
Hall symbol: P 2yb	Cu *K*α radiation, λ = 1.54187 Å
*a* = 10.9397 (1) Å	Cell parameters from 14102 reflections
*b* = 9.4251 (1) Å	θ = 2.9–68.3°
*c* = 15.4833 (10) Å	µ = 2.06 mm^−^^1^
β = 96.414 (7)°	*T* = 123 K
*V* = 1586.46 (10) Å^3^	Block, colourless
*Z* = 4	0.2 × 0.2 × 0.18 mm

### Data collection

**Table:**

Rigaku R-AXIS RAPID II imaging plate diffractometer	4917 independent reflections
Radiation source: fine-focus sealed tube	4440 reflections with *I* > 2σ(*I*)
Graphite monochromator	*R*_int_ = 0.037
Detector resolution: 10.0 pixels mm^-1^	θ_max_ = 65.5°, θ_min_ = 2.9°
ω scans	*h* = −12→12
Absorption correction: multi-scan (*ABSCOR*; Higashi, 1995)	*k* = −8→11
*T*_min_ = 0.55, *T*_max_ = 0.65	*l* = −18→18
14179 measured reflections	

### Refinement

**Table:**

Refinement on *F*^2^	Secondary atom site location: difference Fourier map
Least-squares matrix: full	Hydrogen site location: inferred from neighbouring sites
*R*[*F*^2^ > 2σ(*F*^2^)] = 0.034	H-atom parameters constrained
*wR*(*F*^2^) = 0.087	*w* = 1/[σ^2^(*F*_o_^2^) + (0.0495*P*)^2^] where *P* = (*F*_o_^2^ + 2*F*_c_^2^)/3
*S* = 1.04	(Δ/σ)_max_ < 0.001
4917 reflections	Δρ_max_ = 0.30 e Å^−^^3^
404 parameters	Δρ_min_ = −0.26 e Å^−^^3^
1 restraint	Absolute structure: Flack (1983), 2059 Friedel pairs
Primary atom site location: structure-invariant direct methods	Flack parameter: 0.005 (12)

### Special details

**Table:**

Geometry. All e.s.d.'s (except the e.s.d. in the dihedral angle between two l.s. planes) are estimated using the full covariance matrix. The cell e.s.d.'s are taken into account individually in the estimation of e.s.d.'s in distances, angles and torsion angles; correlations between e.s.d.'s in cell parameters are only used when they are defined by crystal symmetry. An approximate (isotropic) treatment of cell e.s.d.'s is used for estimating e.s.d.'s involving l.s. planes.
Refinement. Refinement of *F*^2^ against ALL reflections. The weighted *R*-factor *wR* and goodness of fit *S* are based on *F*^2^, conventional *R*-factors *R* are based on *F*, with *F* set to zero for negative *F*^2^. The threshold expression of *F*^2^ > σ(*F*^2^) is used only for calculating *R*-factors(gt) *etc*. and is not relevant to the choice of reflections for refinement. *R*-factors based on *F*^2^ are statistically about twice as large as those based on *F*, and *R*- factors based on ALL data will be even larger.

### Fractional atomic coordinates and isotropic or equivalent isotropic displacement parameters (Å^2^)

**Table:**

	*x*	*y*	*z*	*U*_iso_*/*U*_eq_	
S1	0.32715 (6)	0.65201 (7)	0.88376 (4)	0.03365 (17)	
O1	0.25715 (16)	0.2054 (2)	0.92121 (10)	0.0390 (5)	
O2	0.16307 (15)	0.1633 (3)	0.78122 (10)	0.0473 (6)	
O3	0.35387 (15)	0.1347 (2)	0.74148 (10)	0.0349 (4)	
O4	0.32552 (16)	0.49623 (19)	0.92025 (10)	0.0328 (4)	
O5	0.45192 (17)	0.6937 (2)	0.87784 (11)	0.0427 (5)	
O6	0.25396 (18)	0.7302 (2)	0.93686 (11)	0.0473 (5)	
C1	0.2332 (2)	0.1040 (3)	0.85455 (16)	0.0366 (7)	
H1	0.1914	0.0186	0.8760	0.044*	
C2	0.3566 (2)	0.0634 (3)	0.82292 (16)	0.0367 (7)	
H2	0.3673	−0.0415	0.8178	0.044*	
C3	0.4515 (2)	0.1298 (3)	0.88914 (16)	0.0396 (7)	
H3A	0.5227	0.1651	0.8612	0.048*	
H3B	0.4809	0.0607	0.9350	0.048*	
C4	0.3831 (2)	0.2518 (3)	0.92672 (15)	0.0349 (7)	
H4	0.4155	0.2653	0.9892	0.042*	
C5	0.3957 (2)	0.3885 (3)	0.87844 (16)	0.0344 (6)	
H5A	0.4833	0.4164	0.8814	0.041*	
H5B	0.3628	0.3775	0.8166	0.041*	
C6	0.2273 (2)	0.1439 (3)	0.70647 (15)	0.0316 (6)	
C7	0.2104 (3)	0.2734 (3)	0.64975 (17)	0.0452 (7)	
H7A	0.2584	0.2630	0.6004	0.068*	
H7B	0.2385	0.3574	0.6835	0.068*	
H7C	0.1231	0.2841	0.6283	0.068*	
C8	0.1844 (3)	0.0099 (3)	0.65937 (18)	0.0473 (8)	
H8A	0.0972	0.0190	0.6373	0.071*	
H8B	0.1952	−0.0707	0.6995	0.071*	
H8C	0.2327	−0.0059	0.6106	0.071*	
C9	0.2509 (2)	0.6412 (3)	0.77785 (14)	0.0286 (6)	
C10	0.1249 (2)	0.6169 (3)	0.76511 (16)	0.0339 (7)	
H10	0.0797	0.6031	0.8133	0.041*	
C11	0.0667 (2)	0.6132 (3)	0.68185 (16)	0.0346 (7)	
H11	−0.0194	0.5968	0.6731	0.042*	
C12	0.1305 (2)	0.6327 (3)	0.61033 (15)	0.0314 (6)	
C13	0.2563 (2)	0.6543 (3)	0.62421 (14)	0.0304 (6)	
H13	0.3016	0.6666	0.5759	0.037*	
C14	0.3168 (2)	0.6582 (3)	0.70733 (14)	0.0293 (6)	
H14	0.4033	0.6725	0.7161	0.035*	
C15	0.0639 (3)	0.6296 (4)	0.51920 (16)	0.0457 (8)	
H15A	0.1056	0.5634	0.4834	0.069*	
H15B	0.0643	0.7248	0.4938	0.069*	
H15C	−0.0212	0.5985	0.5214	0.069*	
S2	0.37237 (6)	0.33823 (7)	0.40722 (4)	0.03353 (17)	
O11	0.20451 (16)	−0.0793 (2)	0.39873 (10)	0.0370 (5)	
O12	0.15723 (16)	−0.0483 (2)	0.25011 (10)	0.0363 (5)	
O13	0.32440 (15)	−0.1684 (2)	0.22108 (10)	0.0369 (5)	
O14	0.33657 (14)	0.1829 (2)	0.43291 (10)	0.0350 (4)	
O15	0.50001 (15)	0.3417 (2)	0.39625 (11)	0.0383 (5)	
O16	0.32592 (17)	0.4259 (2)	0.47060 (11)	0.0433 (5)	
C21	0.1763 (2)	−0.1485 (3)	0.31885 (15)	0.0373 (7)	
H21	0.1038	−0.2127	0.3199	0.045*	
C22	0.2916 (2)	−0.2316 (3)	0.29894 (15)	0.0356 (7)	
H22	0.2747	−0.3354	0.2916	0.043*	
C23	0.3858 (2)	−0.2014 (3)	0.37619 (16)	0.0397 (7)	
H23A	0.4677	−0.1826	0.3571	0.048*	
H23B	0.3925	−0.2824	0.4172	0.048*	
C24	0.3365 (2)	−0.0699 (3)	0.41833 (16)	0.0328 (6)	
H24	0.3581	−0.0759	0.4827	0.039*	
C25	0.3872 (2)	0.0657 (3)	0.38602 (15)	0.0333 (6)	
H25A	0.4782	0.0657	0.3968	0.040*	
H25B	0.3633	0.0759	0.3228	0.040*	
C26	0.2142 (2)	−0.1047 (3)	0.17900 (15)	0.0352 (7)	
C27	0.2475 (3)	0.0131 (4)	0.12171 (16)	0.0458 (8)	
H27A	0.2835	−0.0264	0.0717	0.069*	
H27B	0.3075	0.0756	0.1545	0.069*	
H27C	0.1736	0.0675	0.1013	0.069*	
C28	0.1314 (3)	−0.2140 (4)	0.13137 (17)	0.0484 (8)	
H28A	0.0541	−0.1691	0.1076	0.073*	
H28B	0.1141	−0.2897	0.1717	0.073*	
H28C	0.1723	−0.2544	0.0838	0.073*	
C29	0.2868 (2)	0.3655 (3)	0.30546 (15)	0.0299 (6)	
C30	0.1609 (2)	0.3732 (3)	0.30083 (16)	0.0380 (7)	
H30	0.1208	0.3655	0.3520	0.046*	
C31	0.0930 (3)	0.3923 (3)	0.22034 (17)	0.0423 (8)	
H31	0.0058	0.3980	0.2168	0.051*	
C32	0.1503 (3)	0.4034 (3)	0.14480 (17)	0.0363 (7)	
C33	0.2781 (3)	0.3983 (3)	0.15257 (17)	0.0397 (7)	
H33	0.3190	0.4075	0.1019	0.048*	
C34	0.3470 (2)	0.3801 (3)	0.23193 (16)	0.0351 (7)	
H34	0.4343	0.3776	0.2361	0.042*	
C35	0.0762 (3)	0.4189 (4)	0.05773 (18)	0.0514 (8)	
H35A	0.1229	0.3808	0.0125	0.077*	
H35B	0.0584	0.5195	0.0462	0.077*	
H35C	−0.0012	0.3665	0.0576	0.077*	

### Atomic displacement parameters (Å^2^)

**Table:**

	*U*^11^	*U*^22^	*U*^33^	*U*^12^	*U*^13^	*U*^23^
S1	0.0464 (4)	0.0303 (4)	0.0239 (3)	−0.0009 (3)	0.0023 (3)	−0.0021 (3)
O1	0.0468 (11)	0.0378 (13)	0.0338 (9)	−0.0049 (9)	0.0105 (8)	−0.0022 (8)
O2	0.0328 (10)	0.0770 (17)	0.0323 (9)	0.0110 (11)	0.0043 (8)	−0.0023 (10)
O3	0.0337 (10)	0.0388 (13)	0.0325 (8)	0.0022 (9)	0.0055 (7)	0.0022 (9)
O4	0.0467 (11)	0.0256 (11)	0.0260 (8)	0.0015 (9)	0.0046 (7)	−0.0009 (8)
O5	0.0482 (11)	0.0449 (14)	0.0326 (9)	−0.0158 (10)	−0.0064 (8)	0.0009 (9)
O6	0.0734 (14)	0.0392 (14)	0.0306 (9)	0.0083 (11)	0.0120 (9)	−0.0024 (9)
C1	0.0457 (16)	0.0346 (19)	0.0308 (13)	−0.0058 (13)	0.0107 (11)	0.0011 (12)
C2	0.0454 (16)	0.0284 (17)	0.0363 (14)	0.0088 (14)	0.0040 (12)	0.0046 (12)
C3	0.0431 (16)	0.035 (2)	0.0391 (14)	0.0106 (13)	−0.0041 (12)	0.0048 (14)
C4	0.0370 (16)	0.0387 (19)	0.0274 (12)	0.0031 (13)	−0.0039 (10)	0.0014 (12)
C5	0.0353 (15)	0.0362 (19)	0.0323 (13)	0.0046 (12)	0.0059 (11)	−0.0011 (12)
C6	0.0322 (14)	0.0336 (18)	0.0289 (12)	−0.0010 (13)	0.0024 (10)	−0.0034 (12)
C7	0.0558 (19)	0.0331 (18)	0.0430 (15)	−0.0011 (15)	−0.0107 (13)	−0.0017 (14)
C8	0.058 (2)	0.038 (2)	0.0438 (15)	−0.0073 (15)	−0.0057 (14)	−0.0009 (14)
C9	0.0312 (13)	0.0279 (16)	0.0266 (11)	0.0024 (12)	0.0031 (10)	0.0003 (12)
C10	0.0319 (14)	0.038 (2)	0.0336 (13)	−0.0029 (12)	0.0112 (11)	0.0061 (12)
C11	0.0233 (13)	0.0341 (19)	0.0465 (15)	−0.0006 (12)	0.0040 (11)	0.0063 (13)
C12	0.0344 (14)	0.0265 (17)	0.0319 (12)	−0.0018 (12)	−0.0028 (11)	−0.0013 (12)
C13	0.0310 (13)	0.0318 (17)	0.0291 (12)	−0.0029 (12)	0.0061 (10)	−0.0002 (12)
C14	0.0250 (13)	0.0357 (17)	0.0273 (11)	−0.0042 (12)	0.0035 (10)	−0.0028 (12)
C15	0.0442 (17)	0.053 (2)	0.0378 (14)	0.0004 (15)	−0.0052 (12)	0.0010 (15)
S2	0.0354 (4)	0.0359 (4)	0.0286 (3)	0.0021 (3)	0.0008 (3)	−0.0048 (3)
O11	0.0358 (10)	0.0478 (14)	0.0283 (9)	0.0019 (9)	0.0081 (7)	−0.0012 (9)
O12	0.0372 (10)	0.0429 (13)	0.0289 (9)	0.0126 (9)	0.0035 (7)	−0.0006 (8)
O13	0.0364 (10)	0.0445 (13)	0.0307 (9)	0.0112 (9)	0.0084 (7)	0.0056 (9)
O14	0.0402 (10)	0.0365 (13)	0.0288 (9)	0.0065 (9)	0.0056 (7)	−0.0015 (8)
O15	0.0312 (10)	0.0436 (13)	0.0390 (10)	−0.0023 (9)	−0.0008 (8)	−0.0065 (9)
O16	0.0512 (12)	0.0435 (14)	0.0352 (10)	0.0072 (10)	0.0049 (8)	−0.0097 (9)
C21	0.0406 (16)	0.0427 (19)	0.0290 (13)	−0.0028 (14)	0.0051 (11)	−0.0005 (13)
C22	0.0446 (17)	0.0300 (17)	0.0331 (13)	0.0048 (13)	0.0084 (11)	0.0057 (12)
C23	0.0458 (17)	0.037 (2)	0.0356 (14)	0.0102 (14)	0.0035 (12)	0.0081 (13)
C24	0.0302 (14)	0.0377 (19)	0.0297 (13)	0.0072 (12)	−0.0002 (10)	0.0043 (12)
C25	0.0329 (14)	0.0381 (18)	0.0290 (13)	0.0079 (13)	0.0037 (10)	−0.0046 (12)
C26	0.0366 (15)	0.0432 (19)	0.0261 (13)	0.0090 (13)	0.0047 (11)	−0.0014 (12)
C27	0.0513 (18)	0.051 (2)	0.0343 (14)	0.0006 (15)	0.0020 (12)	0.0087 (14)
C28	0.0561 (19)	0.055 (2)	0.0329 (14)	−0.0021 (16)	0.0018 (13)	−0.0016 (15)
C29	0.0343 (14)	0.0232 (17)	0.0316 (12)	0.0012 (12)	0.0008 (11)	−0.0029 (11)
C30	0.0312 (15)	0.048 (2)	0.0356 (14)	−0.0004 (13)	0.0085 (11)	0.0047 (13)
C31	0.0286 (15)	0.054 (2)	0.0440 (16)	0.0002 (14)	0.0036 (12)	0.0079 (14)
C32	0.0405 (15)	0.0320 (18)	0.0355 (14)	0.0001 (13)	0.0002 (12)	−0.0003 (12)
C33	0.0420 (17)	0.046 (2)	0.0324 (14)	0.0016 (14)	0.0098 (12)	−0.0005 (13)
C34	0.0275 (14)	0.042 (2)	0.0368 (14)	−0.0018 (12)	0.0059 (11)	−0.0005 (12)
C35	0.055 (2)	0.055 (2)	0.0416 (16)	−0.0039 (17)	−0.0059 (13)	−0.0018 (15)

### Geometric parameters (Å, º)

**Table:**

S1—O6	1.4167 (18)	S2—O16	1.4201 (18)
S1—O5	1.4332 (19)	S2—O15	1.4257 (17)
S1—O4	1.5740 (19)	S2—O14	1.578 (2)
S1—C9	1.758 (2)	S2—C29	1.760 (2)
O1—C1	1.410 (3)	O11—C21	1.402 (3)
O1—C4	1.439 (3)	O11—C24	1.445 (3)
O2—C1	1.413 (3)	O12—C21	1.421 (3)
O2—C6	1.431 (3)	O12—C26	1.427 (3)
O3—C2	1.426 (3)	O13—C22	1.426 (3)
O3—C6	1.432 (3)	O13—C26	1.436 (3)
O4—C5	1.467 (3)	O14—C25	1.464 (3)
C1—C2	1.535 (3)	C21—C22	1.544 (4)
C1—H1	1.0000	C21—H21	1.0000
C2—C3	1.510 (4)	C22—C23	1.516 (4)
C2—H2	1.0000	C22—H22	1.0000
C3—C4	1.522 (4)	C23—C24	1.526 (4)
C3—H3A	0.9900	C23—H23A	0.9900
C3—H3B	0.9900	C23—H23B	0.9900
C4—C5	1.503 (4)	C24—C25	1.501 (4)
C4—H4	1.0000	C24—H24	1.0000
C5—H5A	0.9900	C25—H25A	0.9900
C5—H5B	0.9900	C25—H25B	0.9900
C6—C7	1.502 (4)	C26—C27	1.492 (4)
C6—C8	1.508 (4)	C26—C28	1.509 (4)
C7—H7A	0.9800	C27—H27A	0.9800
C7—H7B	0.9800	C27—H27B	0.9800
C7—H7C	0.9800	C27—H27C	0.9800
C8—H8A	0.9800	C28—H28A	0.9800
C8—H8B	0.9800	C28—H28B	0.9800
C8—H8C	0.9800	C28—H28C	0.9800
C9—C14	1.383 (3)	C29—C30	1.372 (3)
C9—C10	1.390 (3)	C29—C34	1.385 (3)
C10—C11	1.373 (3)	C30—C31	1.390 (4)
C10—H10	0.9500	C30—H30	0.9500
C11—C12	1.385 (3)	C31—C32	1.392 (3)
C11—H11	0.9500	C31—H31	0.9500
C12—C13	1.384 (3)	C32—C33	1.391 (4)
C12—C15	1.514 (3)	C32—C35	1.501 (4)
C13—C14	1.380 (3)	C33—C34	1.378 (4)
C13—H13	0.9500	C33—H33	0.9500
C14—H14	0.9500	C34—H34	0.9500
C15—H15A	0.9800	C35—H35A	0.9800
C15—H15B	0.9800	C35—H35B	0.9800
C15—H15C	0.9800	C35—H35C	0.9800
			
O6—S1—O5	119.90 (13)	O16—S2—O15	119.98 (12)
O6—S1—O4	104.30 (11)	O16—S2—O14	104.35 (11)
O5—S1—O4	109.14 (11)	O15—S2—O14	108.99 (11)
O6—S1—C9	109.36 (12)	O16—S2—C29	109.76 (12)
O5—S1—C9	108.18 (11)	O15—S2—C29	108.69 (11)
O4—S1—C9	104.94 (11)	O14—S2—C29	103.82 (11)
C1—O1—C4	110.36 (19)	C21—O11—C24	109.26 (19)
C1—O2—C6	109.11 (18)	C21—O12—C26	106.85 (19)
C2—O3—C6	106.69 (18)	C22—O13—C26	106.29 (18)
C5—O4—S1	117.38 (15)	C25—O14—S2	117.15 (15)
O1—C1—O2	111.1 (2)	O11—C21—O12	110.5 (2)
O1—C1—C2	107.7 (2)	O11—C21—C22	108.0 (2)
O2—C1—C2	105.09 (18)	O12—C21—C22	104.04 (18)
O1—C1—H1	110.9	O11—C21—H21	111.3
O2—C1—H1	110.9	O12—C21—H21	111.3
C2—C1—H1	110.9	C22—C21—H21	111.3
O3—C2—C3	110.5 (2)	O13—C22—C23	112.1 (2)
O3—C2—C1	103.4 (2)	O13—C22—C21	104.2 (2)
C3—C2—C1	104.0 (2)	C23—C22—C21	104.2 (2)
O3—C2—H2	112.7	O13—C22—H22	112.0
C3—C2—H2	112.7	C23—C22—H22	112.0
C1—C2—H2	112.7	C21—C22—H22	112.0
C2—C3—C4	104.0 (2)	C22—C23—C24	104.4 (2)
C2—C3—H3A	110.9	C22—C23—H23A	110.9
C4—C3—H3A	110.9	C24—C23—H23A	110.9
C2—C3—H3B	110.9	C22—C23—H23B	110.9
C4—C3—H3B	110.9	C24—C23—H23B	110.9
H3A—C3—H3B	109.0	H23A—C23—H23B	108.9
O1—C4—C5	111.8 (2)	O11—C24—C25	112.3 (2)
O1—C4—C3	104.8 (2)	O11—C24—C23	104.6 (2)
C5—C4—C3	112.4 (2)	C25—C24—C23	112.8 (2)
O1—C4—H4	109.2	O11—C24—H24	109.0
C5—C4—H4	109.2	C25—C24—H24	109.0
C3—C4—H4	109.2	C23—C24—H24	109.0
O4—C5—C4	106.98 (19)	O14—C25—C24	107.61 (19)
O4—C5—H5A	110.3	O14—C25—H25A	110.2
C4—C5—H5A	110.3	C24—C25—H25A	110.2
O4—C5—H5B	110.3	O14—C25—H25B	110.2
C4—C5—H5B	110.3	C24—C25—H25B	110.2
H5A—C5—H5B	108.6	H25A—C25—H25B	108.5
O3—C6—O2	104.07 (17)	O12—C26—O13	102.86 (18)
O3—C6—C7	108.8 (2)	O12—C26—C27	109.8 (2)
O2—C6—C7	109.2 (2)	O13—C26—C27	109.4 (2)
O3—C6—C8	111.6 (2)	O12—C26—C28	110.0 (2)
O2—C6—C8	110.1 (2)	O13—C26—C28	111.3 (3)
C7—C6—C8	112.7 (2)	C27—C26—C28	113.0 (2)
C6—C7—H7A	109.5	C26—C27—H27A	109.5
C6—C7—H7B	109.5	C26—C27—H27B	109.5
H7A—C7—H7B	109.5	H27A—C27—H27B	109.5
C6—C7—H7C	109.5	C26—C27—H27C	109.5
H7A—C7—H7C	109.5	H27A—C27—H27C	109.5
H7B—C7—H7C	109.5	H27B—C27—H27C	109.5
C6—C8—H8A	109.5	C26—C28—H28A	109.5
C6—C8—H8B	109.5	C26—C28—H28B	109.5
H8A—C8—H8B	109.5	H28A—C28—H28B	109.5
C6—C8—H8C	109.5	C26—C28—H28C	109.5
H8A—C8—H8C	109.5	H28A—C28—H28C	109.5
H8B—C8—H8C	109.5	H28B—C28—H28C	109.5
C14—C9—C10	120.2 (2)	C30—C29—C34	121.2 (2)
C14—C9—S1	119.69 (18)	C30—C29—S2	119.01 (19)
C10—C9—S1	120.11 (18)	C34—C29—S2	119.74 (19)
C11—C10—C9	119.1 (2)	C29—C30—C31	119.1 (2)
C11—C10—H10	120.5	C29—C30—H30	120.4
C9—C10—H10	120.5	C31—C30—H30	120.4
C10—C11—C12	121.7 (2)	C30—C31—C32	121.1 (2)
C10—C11—H11	119.2	C30—C31—H31	119.4
C12—C11—H11	119.2	C32—C31—H31	119.4
C13—C12—C11	118.4 (2)	C33—C32—C31	117.9 (2)
C13—C12—C15	120.9 (2)	C33—C32—C35	121.2 (2)
C11—C12—C15	120.7 (2)	C31—C32—C35	120.9 (3)
C14—C13—C12	120.9 (2)	C34—C33—C32	121.7 (2)
C14—C13—H13	119.6	C34—C33—H33	119.1
C12—C13—H13	119.6	C32—C33—H33	119.1
C13—C14—C9	119.7 (2)	C33—C34—C29	118.8 (2)
C13—C14—H14	120.2	C33—C34—H34	120.6
C9—C14—H14	120.2	C29—C34—H34	120.6
C12—C15—H15A	109.5	C32—C35—H35A	109.5
C12—C15—H15B	109.5	C32—C35—H35B	109.5
H15A—C15—H15B	109.5	H35A—C35—H35B	109.5
C12—C15—H15C	109.5	C32—C35—H35C	109.5
H15A—C15—H15C	109.5	H35A—C35—H35C	109.5
H15B—C15—H15C	109.5	H35B—C35—H35C	109.5
			
O6—S1—O4—C5	−179.34 (17)	O16—S2—O14—C25	−171.83 (16)
O5—S1—O4—C5	−50.06 (19)	O15—S2—O14—C25	−42.51 (17)
C9—S1—O4—C5	65.69 (18)	C29—S2—O14—C25	73.20 (17)
C4—O1—C1—O2	−105.4 (2)	C24—O11—C21—O12	−94.0 (2)
C4—O1—C1—C2	9.2 (3)	C24—O11—C21—C22	19.2 (3)
C6—O2—C1—O1	124.0 (2)	C26—O12—C21—O11	139.4 (2)
C6—O2—C1—C2	7.8 (3)	C26—O12—C21—C22	23.7 (3)
C6—O3—C2—C3	−140.6 (2)	C26—O13—C22—C23	−135.6 (2)
C6—O3—C2—C1	−29.8 (3)	C26—O13—C22—C21	−23.6 (3)
O1—C1—C2—O3	−105.1 (2)	O11—C21—C22—O13	−117.4 (2)
O2—C1—C2—O3	13.4 (3)	O12—C21—C22—O13	0.1 (3)
O1—C1—C2—C3	10.4 (3)	O11—C21—C22—C23	0.2 (3)
O2—C1—C2—C3	128.9 (2)	O12—C21—C22—C23	117.7 (2)
O3—C2—C3—C4	85.9 (2)	O13—C22—C23—C24	94.0 (2)
C1—C2—C3—C4	−24.6 (3)	C21—C22—C23—C24	−18.0 (3)
C1—O1—C4—C5	97.1 (2)	C21—O11—C24—C25	92.0 (3)
C1—O1—C4—C3	−24.9 (2)	C21—O11—C24—C23	−30.7 (3)
C2—C3—C4—O1	30.4 (3)	C22—C23—C24—O11	29.6 (3)
C2—C3—C4—C5	−91.2 (3)	C22—C23—C24—C25	−92.8 (3)
S1—O4—C5—C4	178.03 (15)	S2—O14—C25—C24	−177.50 (16)
O1—C4—C5—O4	62.0 (3)	O11—C24—C25—O14	63.8 (2)
C3—C4—C5—O4	179.55 (19)	C23—C24—C25—O14	−178.25 (19)
C2—O3—C6—O2	35.0 (3)	C21—O12—C26—O13	−38.7 (3)
C2—O3—C6—C7	151.4 (2)	C21—O12—C26—C27	−155.1 (2)
C2—O3—C6—C8	−83.7 (3)	C21—O12—C26—C28	79.9 (3)
C1—O2—C6—O3	−26.1 (3)	C22—O13—C26—O12	38.6 (3)
C1—O2—C6—C7	−142.1 (2)	C22—O13—C26—C27	155.2 (2)
C1—O2—C6—C8	93.7 (3)	C22—O13—C26—C28	−79.2 (2)
O6—S1—C9—C14	138.3 (2)	O16—S2—C29—C30	−43.9 (3)
O5—S1—C9—C14	6.1 (3)	O15—S2—C29—C30	−176.9 (2)
O4—S1—C9—C14	−110.3 (2)	O14—S2—C29—C30	67.2 (2)
O6—S1—C9—C10	−41.2 (3)	O16—S2—C29—C34	135.5 (2)
O5—S1—C9—C10	−173.4 (2)	O15—S2—C29—C34	2.5 (3)
O4—S1—C9—C10	70.2 (2)	O14—S2—C29—C34	−113.4 (2)
C14—C9—C10—C11	−1.4 (4)	C34—C29—C30—C31	1.7 (4)
S1—C9—C10—C11	178.1 (2)	S2—C29—C30—C31	−178.9 (2)
C9—C10—C11—C12	0.1 (4)	C29—C30—C31—C32	0.2 (5)
C10—C11—C12—C13	1.0 (4)	C30—C31—C32—C33	−1.6 (5)
C10—C11—C12—C15	−179.3 (3)	C30—C31—C32—C35	177.8 (3)
C11—C12—C13—C14	−0.8 (4)	C31—C32—C33—C34	1.2 (5)
C15—C12—C13—C14	179.5 (3)	C35—C32—C33—C34	−178.1 (3)
C12—C13—C14—C9	−0.4 (4)	C32—C33—C34—C29	0.5 (4)
C10—C9—C14—C13	1.6 (4)	C30—C29—C34—C33	−2.0 (4)
S1—C9—C14—C13	−177.9 (2)	S2—C29—C34—C33	178.6 (2)

### Hydrogen-bond geometry (Å, º)

**Table:**

*D*—H···*A*	*D*—H	H···*A*	*D*···*A*	*D*—H···*A*
C4—H4···O5^i^	1.00	2.48	3.390 (3)	152
C5—H5*B*···O3	0.99	2.56	3.196 (3)	122
C11—H11···O12^ii^	0.95	2.44	3.163 (3)	133
C14—H14···O5	0.95	2.51	2.897 (3)	105
C24—H24···O15^iii^	1.00	2.42	3.315 (3)	148
C28—H28*C*···O6^iv^	0.98	2.54	3.471 (3)	159
C34—H34···O15	0.95	2.53	2.908 (3)	104

Symmetry codes: (i) −*x*+1, *y*−1/2, −*z*+2; (ii) −*x*, *y*+1/2, −*z*+1; (iii) −*x*+1, *y*−1/2, −*z*+1; (iv) *x*, *y*−1, *z*−1.
